# Inhibition of Pro-inflammatory and Anti-apoptotic Biomarkers during Experimental Oral Cancer Chemoprevention by Dietary Black Raspberries

**DOI:** 10.3389/fimmu.2017.01325

**Published:** 2017-10-23

**Authors:** Steve Oghumu, Bruce C. Casto, Jennifer Ahn-Jarvis, Logan C. Weghorst, Jim Maloney, Paul Geuy, Kyle Z. Horvath, Claire E. Bollinger, Blake M. Warner, Kurt F. Summersgill, Christopher M. Weghorst, Thomas J. Knobloch

**Affiliations:** ^1^Division of Environmental Health Sciences, College of Public Health, The Ohio State University Columbus, Columbus, OH, United States; ^2^Comprehensive Cancer Center, The Ohio State University Columbus, Columbus, OH, United States; ^3^School of Dental Medicine, University of Pittsburgh, Pittsburgh, PA, United States; ^4^Department of Otolaryngology, College of Medicine, The Ohio State University Columbus, Columbus, OH, United States

**Keywords:** oral cancer, black raspberry, chemoprevention, pro-inflammatory, biomarker

## Abstract

Oral cancer continues to be a significant public health problem worldwide. Recently conducted clinical trials demonstrate the ability of black raspberries (BRBs) to modulate biomarkers of molecular efficacy that supports a chemopreventive strategy against oral cancer. However, it is essential that a preclinical animal model of black raspberry (BRB) chemoprevention which recapitulates human oral carcinogenesis be developed, so that we can validate biomarkers and evaluate potential mechanisms of action. We therefore established the ability of BRBs to inhibit oral lesion formation in a carcinogen-induced rat oral cancer model and examined potential mechanisms. F344 rats were administered 4-nitroquinoline 1-oxide (4NQO) (20 µg/ml) in drinking water for 14 weeks followed by regular drinking water for 6 weeks. At week 14, rats were fed a diet containing either 5 or 10% BRB, or 0.4% ellagic acid (EA), a BRB phytochemical. Dietary administration of 5 and 10% BRB reduced oral lesion incidence and multiplicity by 39.3 and 28.6%, respectively. Histopathological analyses demonstrate the ability of BRBs and, to a lesser extent EA, to inhibit the progression of oral cancer. Oral lesion inhibition by BRBs was associated with a reduction in the mRNA expression of pro-inflammatory biomarkers *Cxcl1, Mif*, and *Nfe2l2* as well as the anti-apoptotic and cell cycle associated markers *Birc5, Aurka, Ccna1*, and *Ccna2*. Cellular proliferation (Ki-67 staining) in tongue lesions was inhibited by BRBs and EA. Our study demonstrates that, in the rat 4NQO oral cancer model, dietary administration of BRBs inhibits oral carcinogenesis via inhibition of pro-inflammatory and anti-apoptotic pathways.

## Introduction

It is estimated that there are about 48,330 new cases and 9,570 deaths due to oral cancer in the US annually (1). This amounts to greater than one person every hour every day that dies from oral cancer. Worldwide, about 300,000 cases and 145,000 deaths are reported annually (2). Due to epithelial field defects associated with oral carcinogenesis, tumor recurrence and second primary tumor incidence are common. Despite advances in oral cancer treatment strategies, survival rates have not significantly improved over the past three decades. Consequently, new and effective approaches to oral cancer prevention and therapy are needed, and it is essential that these strategies be developed and tested in preclinical models that reflect essential features of human oral carcinogenesis.

As the molecular mechanisms that drive the multistep process of oral carcinogenesis are becoming better understood, inhibitors that target oncogenic pathways have been used in oral cancer chemoprevention and treatment. Agents used in oral cancer preclinical and clinical chemoprevention studies include inhibitors of cyclooxygenase 2 (Cox-2) (3, 4) and epidermal growth factor receptor (EGFR) (5). However, toxicity associated with molecular targeted therapeutic approaches are common.

Epidemiologic and case–control studies demonstrate a consistent inverse correlation between increased fruit and vegetable consumption and decreased oral cancer risk (6, 7). In support of these data, recent preclinical studies have clearly demonstrated the remarkable ability of black raspberries (BRBs) to prevent tumor development in oral cancer cells *in vitro* (8, 9), as well as in animal models of oral cancer in hamsters (10, 11), and esophageal and colon cancers in rats (12–14). BRBs possess a number of bioactive phytochemicals such as anthocyanins, ellagitannins, ellagic acid (EA), and others which may act in an additive or synergistic manner to inhibit cancer development. Recent clinical studies conducted by our group demonstrate the ability of dietary black raspberry (BRB) administration to modulate molecular biomarkers within the oral cavity in a manner that supports a chemopreventive strategy for oral cancer (15). These biomarkers include genes affecting inflammatory and apoptotic pathways which are crucial for oral cancer initiation, promotion, and progression (15). To translate clinically derived molecular signature of BRB responsive biomarkers into defined mechanisms of action, an appropriate experimental system of oral carcinogenesis is needed.

One of the most common animal models of oral cancer chemoprevention is the 7,12-dimethlybenz(a)anthracene (DMBA) induced hamster cheek pouch model which reflects elements characterizing human oral carcinogenesis. We have previously published on the chemopreventive efficacy of BRBs using this model (10, 11). However, this model lacks the curated and annotated genomic infrastructure necessary for the interrogation of human-relevant molecular biomarkers and the elucidation of cellular and molecular mechanisms of action. An animal model which recapitulates fundamental features of human oral carcinogenesis, is amenable to chemopreventive intervention, and has established tools for cellular, molecular, and systems biology interrogation is required for evaluation of oral cancer chemoprevention mechanistic studies. The carcinogen-induced rat oral cancer model fulfills these criteria and was therefore used in this study.

The objective of this study was to (i) establish and validate a preclinical model of oral cancer chemoprevention by BRBs which recapitulates the clinical and molecular features of human disease and (ii) define primary mechanisms of action of oral chemoprevention by the bioactive phytochemicals in BRBs using this preclinical model. To accomplish these objectives, we used 4-nitroquinoline 1-oxide (4NQO) to initiate oral carcinogenesis in F344 rats, a model that has been widely used in experimental oral carcinogenesis and chemoprevention studies (16–18). Our results demonstrate that experimental rat oral carcinogenesis represents an essential tool for the extended investigation of BRB-mediated oral chemoprevention, and validate the involvement of inflammatory, apoptotic, and cell cycle associated pathways in the oral cancer inhibitory activity of BRBs.

## Materials and Methods

### Animals

Male F344 rats, 6–7 weeks old were obtained from Harlan Laboratories (Indianapolis, IN, USA) and housed at an HEPA-filtered animal facility at The Ohio State University according to animal protocols and regulations of the University Laboratory Animal Resources (PHS Animal Welfare Assurance #A3261-01 and Association for Assessment and Accreditation of Laboratory Animal Care International #0028). All experiments with rats were approved by The Ohio State University Institutional Animal Care and Use Committee (Protocol #2010A00000085) and Institutional Biosafety Committee.

### Chemicals

The carcinogen 4NQO was purchased from Sigma-Aldrich (St. Louis, MO, USA; #N8141) and stored in foil-wrapped containers at −20°C. Fresh 4NQO solutions (20 µg/ml in drinking water) were prepared twice weekly for administration to rats for 14 weeks. BRBs (*Rubus occidentalis* “Jewel variety”) were obtained from the Stokes Berry Farm (Wilmington, OH, USA) and shipped frozen to Van Drunen Farms (Momence, IL, USA) for freeze drying. BRB powder was stored at −20°C until incorporated into custom purified AIN-76A animal diet pellets at 5 and 10% w/w concentrations (Dyets, Inc., Bethlehem, PA, USA). EA was purchased from Sigma-Aldrich (St. Louis, MO, USA; #E2250, ≥95% HPLC) and incorporated into AIN-76A pellets at 0.4%, proportionally matched to the amount present in 10% lyophilized BRBs (Oregon Raspberry & Blackberry Commission; Corvallis, OR, USA).

### Rat Oral Carcinogenesis and Chemoprevention

**Figure 1:**
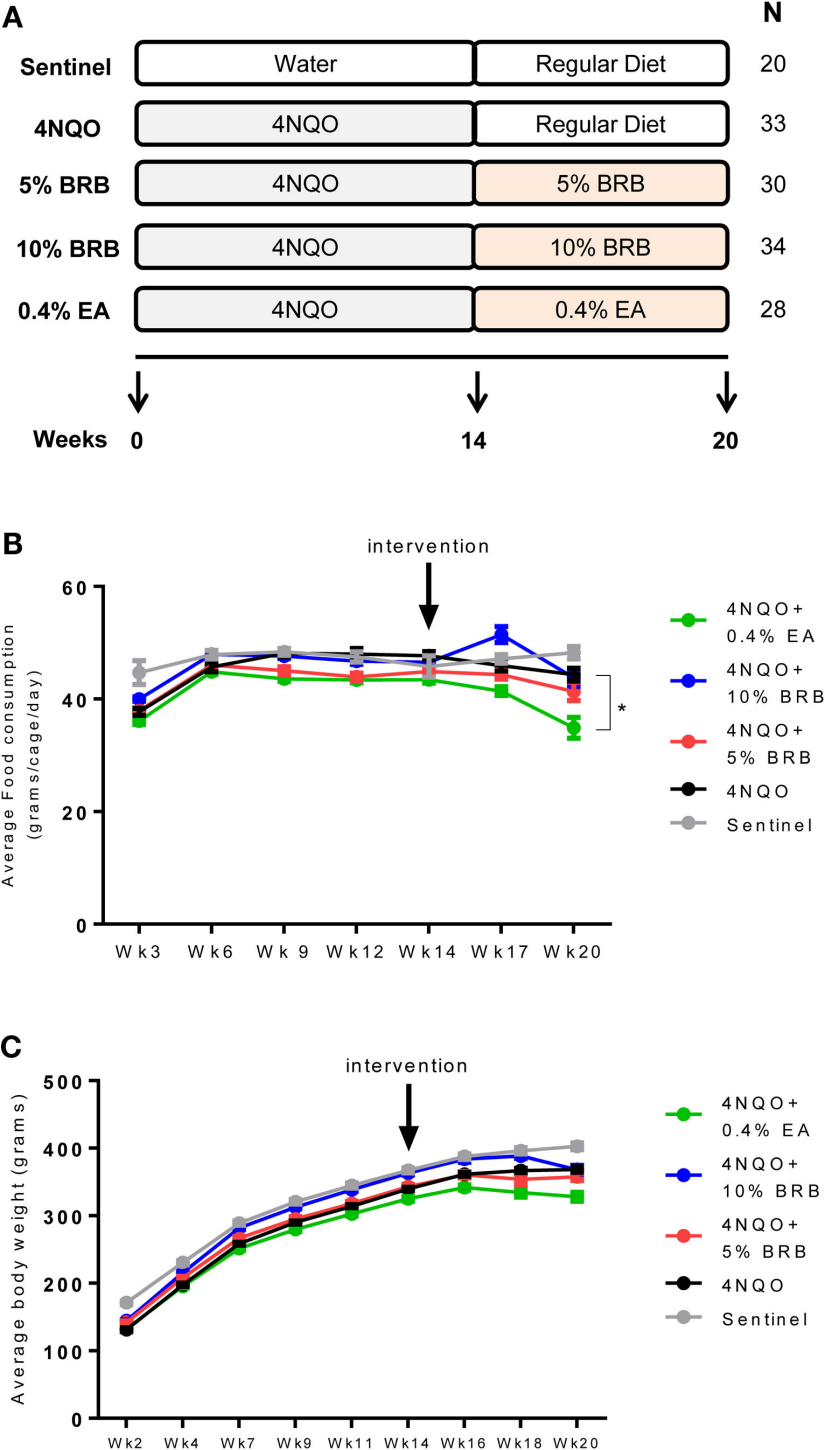
**(A)** Scheme of rat oral carcinogenesis and chemoprevention. Rats were exposed to oral carcinogen 4-nitroquinoline 1-oxide (4NQO) (20 μg/ml in drinking water) for 14 weeks. Administration of dietary chemopreventive agent began at week 14 and continued for 6 weeks. Animals were sacrificed at week 20 for gross and histological analysis of tongue lesions and examination of cellular and molecular markers of carcinogenesis. *N* represents number of rats per group. **(B)** Average food consumption for the control and experimental rat groups during oral carcinogenesis and dietary intervention phases of the experimental protocol. Data are expressed as mean food consumption (grams/cage/day) ± SE in each rat cage belonging to an experimental group (three rats per cage). *p-value < 0.05 for food intake comparison between 4NQO-only rat group and 4NQO + ellagic acid (EA) rat group using independent Student’s *t*-test. **(C)** Average body weight measurements for control and experimental rat groups during the carcinogenesis and intervention phases of the experimental protocol. With the exception of the sentinel animals, all experimental animals were administered 4NQO and were on AIN-76A defined diet prior to intervention with dietary black raspberries (BRBs) or EA. Data are expressed as mean ± SE of all rats in the group.

A control sentinel group (Group 1, *N* = 20) received regular drinking water without carcinogen (4NQO) and fed unmodified AIN-76A diet. Rats belonging to experimental groups were administered 4NQO in drinking water for 14 weeks, then randomized into four groups (Groups 2–5, *N* = 35 per group) for 6 weeks of chemopreventive agent administration (Figure 1A). Group 2 (carcinogen control group) received unmodified AIN-76A diet without chemopreventive agent, Group 3 received AIN-76A diet containing 5% BRB, Group 4 received AIN-76A diet containing 10% BRB, and Group 5 received AIN-76A diet containing 0.4% EA. After a 14-week carcinogen exposure and 6-week chemopreventive agent administration (20-week protocol), animals were sacrificed. Rat tongues were harvested and gross lesions were counted, categorized, and recorded. Tongue tissues were fixed in 10% neutral buffered formalin, paraffin embedded, and sectioned for histopathology and immunohistochemistry (IHC). Total RNA was prepared from tongue tissues for reverse transcription quantitative PCR (RT-qPCR) analysis. Whole blood samples were obtained from each animal for enzyme-linked immunosorbent assay (ELISA).

### Histopathology Calibration and Grading of Microscopic Lesions

Whole rat tongues were cut four times longitudinally into equal width sections. One half (two pieces, medial and lateral sections) of the tongues were placed on edge into tissue cassettes and fixed in formalin for 24 h. After fixation, the tissues were embedded in paraffin and three 5 µm sections of each tissue block were cut and mounted on Superfrost Plus slides (Fisher Scientific, Pittsburgh, PA, USA). Hematoxylin and eosin (H&E) stained slides were prepared from each block. Each section and level of tongue was delineated into three equal regions (anterior, middle, and posterior tongue) along the surface of the tongue epithelia using a millimeter ruler and dotting pen. Before grading the slides, a calibration set (*N* = 10) was selected from the set of H&E slides using a random number generator. The calibration set was reviewed independently by two oral and maxillofacial pathologists and the results were reviewed together at the microscope. Differences in grading were discussed and agreement was achieved. Blinded to the treatment groupings of the animals, the highest histopathological grade of each of the two pieces of tongue, within each anatomic region of the tongue (anterior, middle, posterior), for each section was graded independently by the oral and maxillofacial pathologists. As with the calibration set, differences in grading were discussed and agreement was achieved. The four histological categories used for grading were normal, low-grade dysplasia, high-grade dysplasia, or squamous cell carcinoma, as described previously (11). Low-grade dysplasia was characterized by changes in the epithelium such as basilar crowding and hyperplasia, cellular disorganization, and maturational disturbances within the lower one-third to the middle third of the epithelial thickness with little interruption of the keratin layer. High-grade dysplasia included the above parameters extending into the upper third of the epithelial thickness. Additional features included frequent mitotic figures, cellular pleomorphism, nuclear atypia, and some early disturbance of the keratin layer. Further, full thickness epithelial change with the above features, an expansion of multiple layers of cells into the suprabasal and intermediate layers, and with disturbance of the keratin layer but without penetration of the basement membrane was included in this category. Squamous cell carcinoma was defined as the above changes with invasion through the basement membrane.

### Immunohistochemistry

Immunohistochemistry staining was performed on deparaffinized tongue tissue sections by The Ohio State University Comparative Pathology and Mouse Phenotyping Shared Resource, using Ki-67 antibody (1:100 primary antibody) or antibody against cleaved caspase 3 (1:180 primary antibody). Slides were counterstained with hematoxylin. For Ki-67 staining, cell proliferation was determined by amount of Ki-67 positive nuclei relative to total amount of nucleated cells in six random fields of tongue epithelium using a Zeiss Axioplan imaging microscope at 200× magnification and AxioVision Imaging software (Carl Zeiss Microscopy, LLC, Thornwood, NY, USA).

### Reverse Transcription Quantitative PCR

Total RNA was extracted from rat tongue tissues using the AllPrep DNA/RNA Kit (Qiagen, Valencia, CA, USA). RNA was reverse transcribed to cDNA using the High Capacity cDNA Reverse Transcription Kit (Applied Biosystems, Foster City, CA, USA). PCR amplification was performed (*N* ≥ 20 rats per group) using pre-validated rat-specific TaqMan Assays (Applied Biosystems, Foster City, CA, USA) in duplicates. Comparative Cq estimates were performed after reference gene set normalization. The reference gene normalization was determined using the qbase + real-time PCR data analysis software (19, 20) (Biogazelle, Ghent, Belgium), which selects the most stably expressed genes across all rat tongue RNA samples in all groups. For TaqMan assays, optimal reference genes were determined by qbase + geNorm software (19, 20) to be the combination of *Egfr* and *Bcl2*. In some experiments, rat primer sequences were selected using the Real-Time qPCR Assay tool (https://www.idtdna.com/scitools/Applications/RealTimePCR/) by Integrated DNA Technologies (Coralville, IA, USA), and PCR amplification was performed using SYBR Green chemistry (Qiagen, Valencia, CA, USA). For SYBR Green assays, optimal reference genes were determined by qbase + geNorm software (19, 20) to be the combination of *Gapdh* and *Tgfb*. In both cases, reference genes were calculated to be of high reference target stability. Genes targeted included interleukin 1 beta (*Il-1β)*, nuclear factor kappa B subunit 1 *(Nfkb1)*, arginase 1 (*Arg1)*, prostaglandin-endoperoxidase synthase 1 (*Ptgs1)*, Prostaglandin-endoperoxidase synthase 2 (*Ptgs2*), C-X-C motif chemokine ligand 1 (*Cxcl1)*, macrophage migration inhibitory factor (*Mif)*, nuclear factor, erythroid 2 like 2 (*Nfe2l2)*, baculoviral IAP repeat containing 5 (*Birc5)*, aurora kinase A (*Aurka*), cyclin A1 (*Ccna1*), cyclin A2 (*Ccna2*), caspase 14 (*Casp14)*.

### Enzyme-Linked Immunosorbent Assay

Quantitative detection of PTGS1/COX-1 in Rat serum was performed using Rat PTGS1/COX1 Sandwich ELISA Kit (#LS-F12318, LifeSpan Biosciences; Seattle, WA, USA). Detection of PTGS2/COX-2 was performed using the Rat COX-2 Competitive ELISA Kit (#RC0087, NeoScientific, Cambridge, MA, USA). Plates were read at an absorbance of 450 nm using Spectramax M3 microplate reader (Molecular Devices LLC, Downingtown, PA, USA) and concentrations were determined by extrapolation from standard curves generated by the Softmax Pro software (Molecular Devices LLC, Downingtown, PA, USA) using the COX-1 and COX-2 standards provided by each respective kit.

### Statistical Analysis

Statistical evaluation was performed using IBM SPSS software Version 24 (Chicago, IL, USA), or Prism 5 (GraphPad Software; San Diego, CA, USA) and *p*-values ≤ 0.05 were considered to be significant. Mean ± SEM was used to report findings. Significant differences (*p*-value < 0.05) between 4NQO and treatment groups (5% BRB, 10% BRB, and 0.4% EA) were discriminated using a Student’s independent *t*-test for lesion endpoint, ELISA, and Ki-67 immunohistochemical analyses. An analysis of variance was used to model the gene expression data with respect to treatment using qbase + real-time PCR data analysis software (19) (Biogazelle, Ghent, Belgium). If discriminant differences were found, the Tukey–Kramer *post hoc* test was used.

## Results

### Effect of BRB Consumption on Food Intake, Weight, and Overall Survival of Carcinogen-Induced Rats

Following exposure to 4NQO in drinking water and subsequent administration of control or experimental diet (Figure 1A), food consumption and body weight measurements were determined for the various groups during the carcinogenesis and intervention phase of our study. Food consumption analysis during the carcinogenesis phase compared sentinel non-exposed animals with all animals that were exposed to the carcinogen 4NQO. No significant changes in food consumption were observed between rats administered 4NQO and non-4NQO sentinel animals (Figure 1B). During the intervention phase, food consumption analysis for each dietary group compared to 4NQO-only control group showed no significant differences in animals fed with 5 and 10% BRB diets. However, a significant reduction in average food consumption was observed in animals fed with 0.4% EA compared to the 4NQO control group (Figure 1B). Average body weights were also recorded for each animal group over the course of the study. We observed no significant changes in body weight between the sentinel group and the pooled group of animals exposed to 4NQO (Figure 1C). However, body weight comparisons between the 4NQO-only control group with the other treatment groups during the intervention phase revealed a significant reduction in body weight in 0.4% EA treated animals compared to 4NQO control animals but no significant body weight changes between 4NQO-only group and 10% BRB treatment group (Figure 1C). Taken together, while there appears to be slightly reduced preference for the 0.4% EA diet, our data demonstrate the negligible effects of whole BRB diet on overall food consumption and body weight of oral carcinogen-induced rats, which suggests that BRB-mediated chemopreventive approaches to oral cancer management are relatively safe with minimal side effects. This agrees with other studies demonstrating that BRB exerts minimal effects on normal oral tissue (21, 22).

### Dietary Administration of BRBs Reduces Oral Lesion Incidence and Multiplicity in 4NQO-Induced Rats

**Figure 2:**
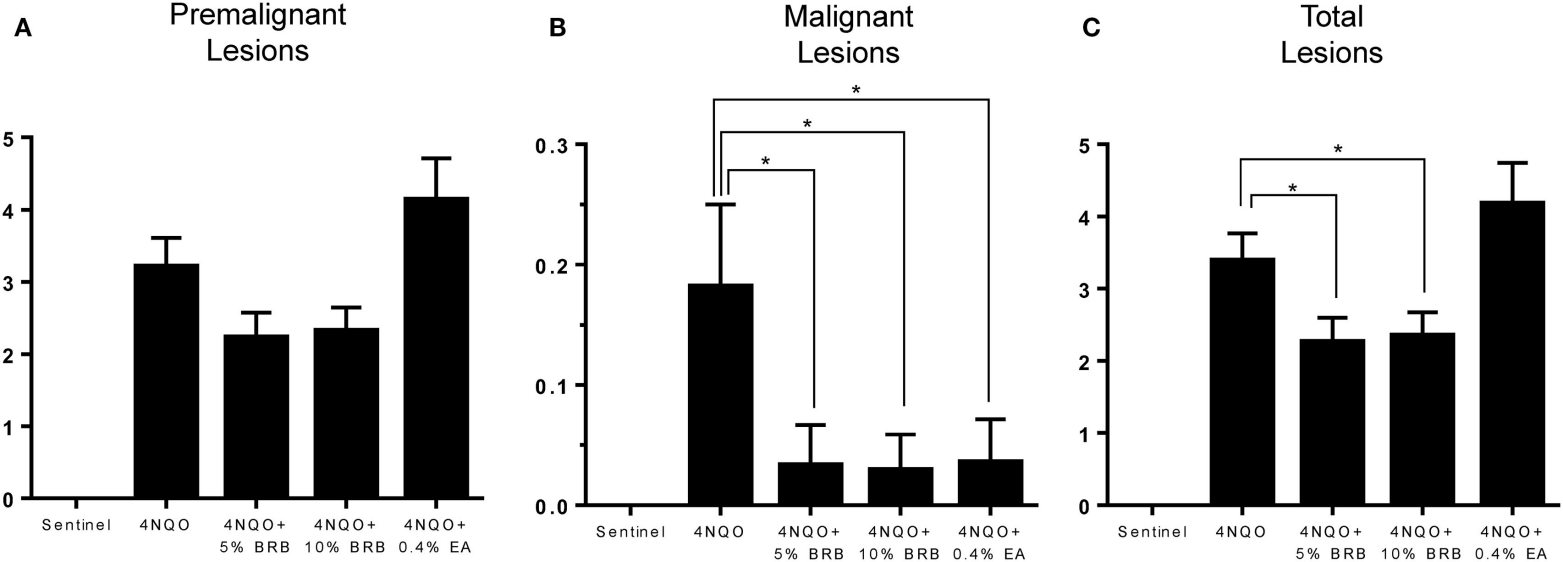
**(A–C)** Average number of **(A)** premalignant, **(B)** malignant, and **(C)** total lesions in 4-nitroquinoline 1-oxide (4NQO)-induced rat tongues fed control diet (4NQO group) or diet supplemented with 5% black raspberry (BRB) (4NQO + 5% BRB group), 10% BRB (4NQO + 10% BRB group), or 0.4% ellagic acid (EA) (4NQO + 0.4% EA group). Data are expressed as mean ± SE of all rats in the group. *p-value < 0.05 for group comparisons using independent Student’s *t*-test.

Next, we examined effects of BRB administration on oral tumor incidence and multiplicity as well as total oral lesion incidence and multiplicity following 4NQO-induced rat oral carcinogenesis. Rats from all experimental groups were sacrificed and characterized for gross lesions, which included premalignant lesions (leukoplakia, erythroplakia), and malignant lesions (overt tumors). These data are summarized in Table 1. Our results indicate that the freeze-dried whole BRB supplemented diets significantly inhibited oral carcinogenesis as evidenced by reduced numbers of tumors as well as combined oral lesions (Figures 2A–C). These effects manifested as reductions in tumor and collective lesion incidence/multiplicity and correspond to 39.3 and 28.6% inhibition of gross lesions with 5 and 10% BRB diets, respectively. Further, both the 5% BRB and 10% BRB groups demonstrated the greatest number of lesion-free animals following 4NQO exposure and intervention, and all dietary interventions showed greater than fivefold decrease in tumor incidence.

**Table 1:** Analysis of gross lesions in tongues of rats induced with the oral carcinogen 4-nitroquinoline 1-oxide (4NQO), with or without dietary administration of chemopreventive agent.

Group	Survival	Tumor incidence	Tumor multiplicity	Lesion incidence	Lesion multiplicity
4NQO	33/35 (94.3%)	6/33 (18.2%)	0.18	30/33 (90.9%)	3.39
4NQO + 5% black raspberries (BRB)	30/35 (85.7%)	1/30 (3.3%)	0.03	23/30 (76.7%)	2.27
4NQO + 10% BRB	34/35 (97.1%)	1/34 (2.9%)	0.03	28/34 (82.4%)	2.35
4NQO + 0.4% ellagic acid	28/35 (80.0%)	1/28 (3.6%)	0.03	24/28 (85.7%)	4.18

**Figure 3:**
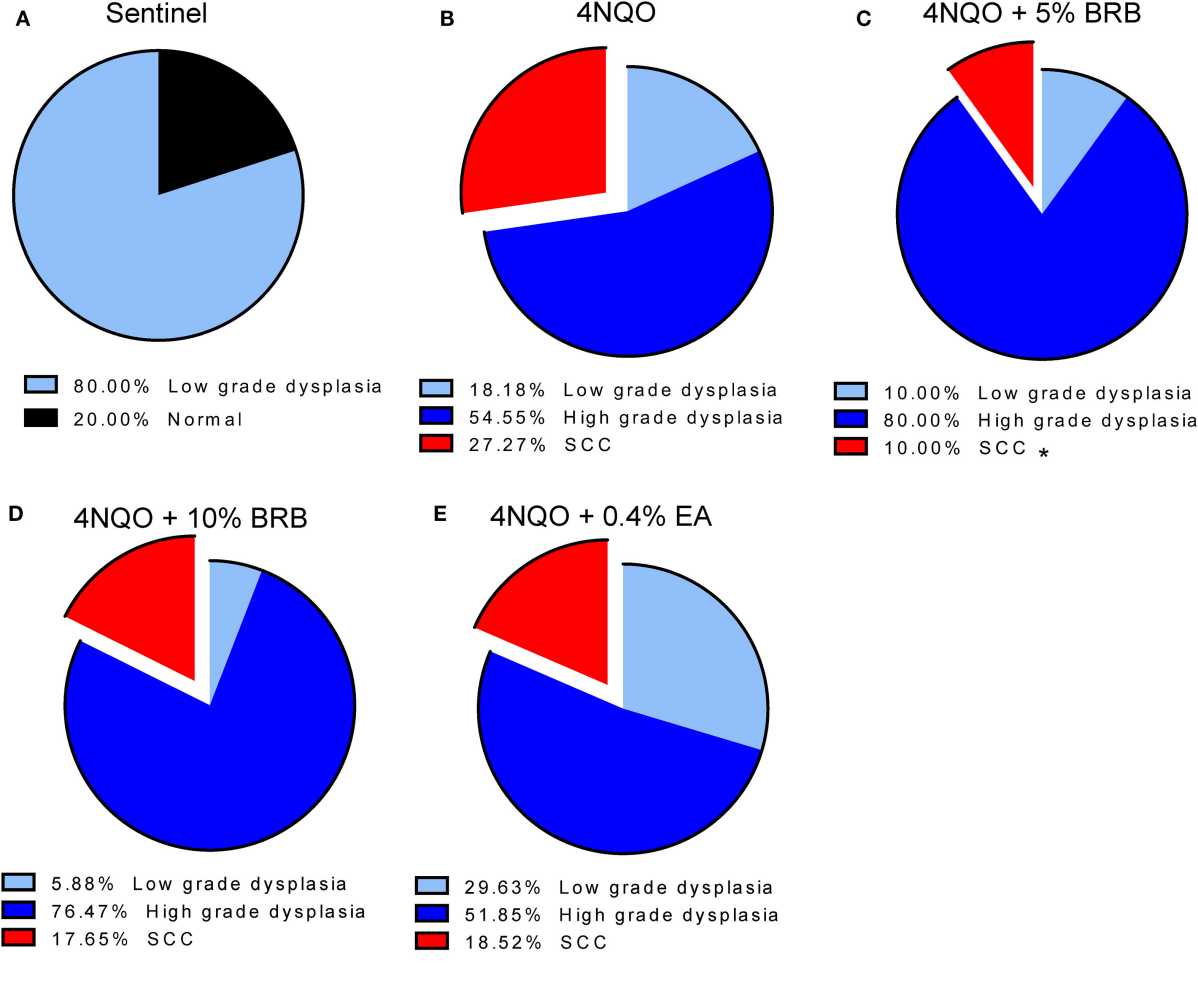
**(A–E)** Histopathological analysis of tongue tissues from untreated and treated carcinogen-induced rats. Distribution of the histopathological grades (normal, low-grade dysplasia, high-grade dysplasia, and SCC) in tongue epithelia of **(A)** sentinel rats, **(B)** 4-nitroquinoline 1-oxide (4NQO)-induced rats fed control diet, **(C)** 4NQO-induced rats fed diet supplemented with 5% black raspberry (BRB), **(D)** 4NQO-induced rats fed diet supplemented with 10% BRB, and **(E)** 4NQO-induced rats fed diet supplemented with 0.4% ellagic acid (EA). Note the significantly reduced percentage of SCCs in 4NQO exposed rats treated with 5% BRB. *p-value < 0.05 for SCC incidence comparison between 4NQO-only exposed rats and 4NQO + 5% BRB rats, using independent Student’s *t*-test.

Histopathological analysis of tongue sections from 4NQO-induced rats further demonstrates the chemopreventive effect of BRB diet against oral carcinogenesis (Figures 3A–E). 5% BRBs significantly reduced the progression of tongue lesions to squamous cell carcinomas as determined by histological grading (Figure 3C). It appears from our data that BRB administration delays the progression of tongue lesions beyond high-grade dysplasia, which is probably associated with the time of administration of chemopreventive agent, which began 14 weeks after 4NQO exposure. It is likely that an earlier intervention with BRBs would result in an even greater inhibition of dysplastic lesions and SCCs.

Comparisons of BRB efficacy between the 5 and 10% BRB diets further demonstrated that 5% BRB administration was more effective than 10% BRB in inhibiting gross lesion incidence and multiplicity. Total numbers of oral premalignant lesions were fewer in 5% BRB administered rats compared to 10% BRB administered rats (Table 1). Similarly, histopathological analysis demonstrated the increased efficacy of 5% BRB compared to 10% BRB at inhibiting SCC development during rat oral carcinogenesis (Figures 3B–D).

### Inhibition of Oral Lesions by BRB Is Associated with Reduction in Pro-inflammatory Markers of Oral Carcinogenesis

**Figure 4:**
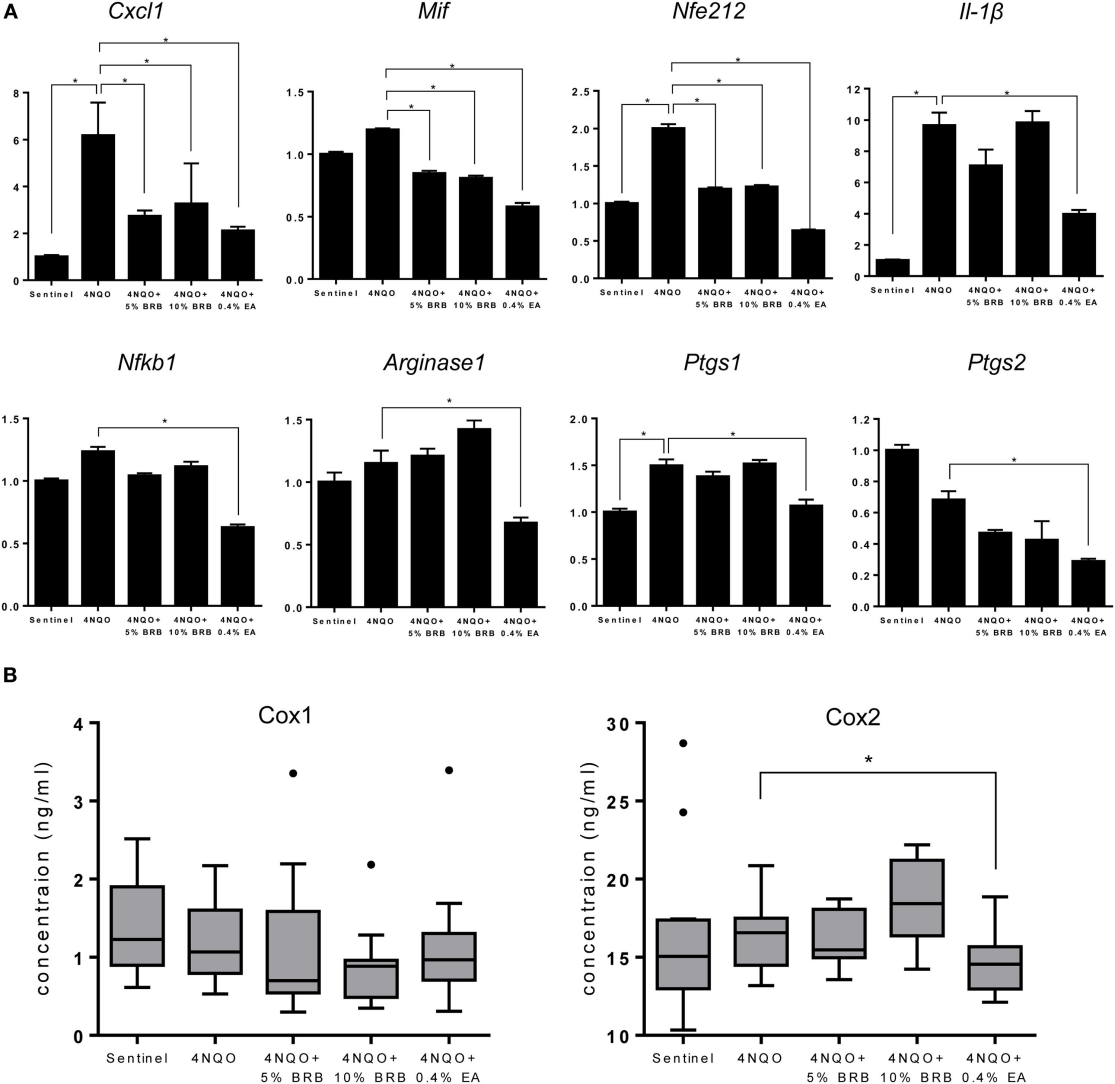
**(A)** Gene expression profiles of the pro-inflammatory markers *Cxcl1, Mif, Nfe212, Il-1β, Nfkb1, Arg1, Ptgs1*, and *Ptgs2* during 4-nitroquinoline 1-oxide (4NQO)-induced oral carcinogenesis determined by reverse transcription quantitative PCR. Data are presented as mean fold induction over sentinels ± SE. *p-value < 0.05 for each group (*N* ≥ 20) comparison using analysis of variance analysis. **(B)** Serum concentrations of cyclooxygenase 1 (Cox-1) and Cox-2 in randomly selected rats from each experimental group (*N* = 15) during 4NQO-induced oral carcinogenesis as determined by enzyme-linked immunosorbent assay. Data are presented as mean ± SE. *p-value < 0.05 for each respective group comparison using independent Student’s *t*-test.

Oral carcinogenesis is characterized by a chronic pro-inflammatory microenvironment, favoring tumor initiation and progression (23, 24). Chronic inflammation is an important underlying factor which promotes carcinogenesis (25). A recent short-term Phase 0 clinical study conducted by our group demonstrated the ability of a BRB troche to inhibit gene expression of pro-inflammatory biomarkers in oral cancer patients (15). We therefore evaluated the ability of BRBs to inhibit pro-inflammatory gene expression profiles in experimental rat oral carcinogenesis induced by 4NQO. Gene expression of the pro-inflammatory biomarkers *Cxcl1, Mif*, and *Nfe2l2* were downregulated in carcinogen-induced rats fed with 5 or 10% BRB diet (Figure 4A). These genes have been shown to play a role in oral carcinogenesis in preclinical models as well as in human oral cancer (26, 27). Prostaglandin-endoperoxidase synthase, a key enzyme in prostaglandin biosynthesis, is especially critical in mediating the pathologic consequences of chronic inflammation in OSCC (23). We therefore examined oral epithelia mRNA expression levels of *Ptgs1* and *Ptgs2*, as well as serum levels of Cox1/Ptgs1 and Cox2/Ptgs2 in normal rats, and in carcinogen-induced rats fed with regular diet, 5 or 10% BRB supplemented diets. However, we observed no significant differences in gene expression levels in rat tongue (Figure 4A) or protein expression levels in rat serum (Figure 4B) of Ptgs1 and Ptgs2 in BRB administered carcinogen-induced rats compared to untreated carcinogen-induced rats. Taken together, our data suggest that a potential mechanism of oral cancer chemoprevention by BRB phytochemicals is the inhibition of specific pro-inflammatory signaling pathways.

### BRBs Inhibit Anti-apoptotic and Proliferative Pathways in 4NQO-Induced Rat Oral Carcinogenesis

**Figure 5:**
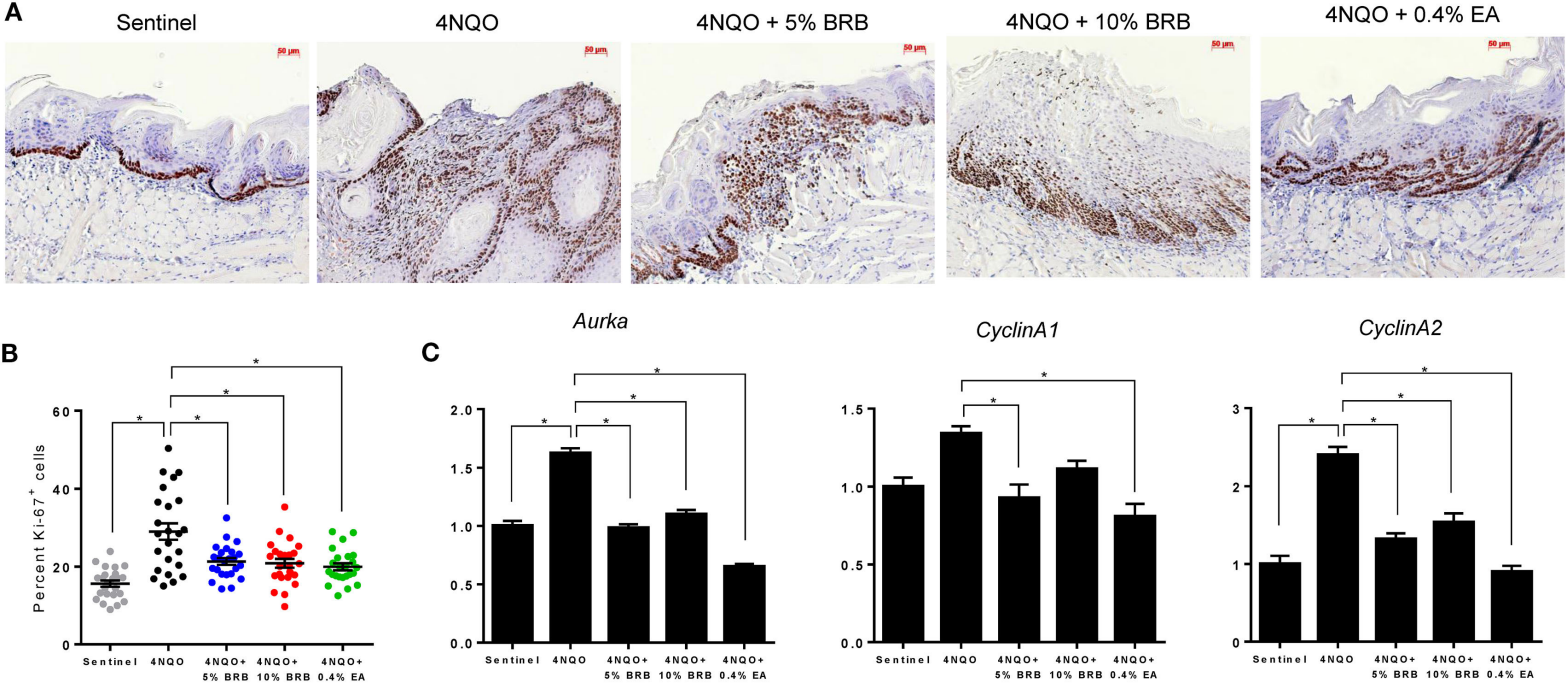
**(A)** Representative microscopic images (×200) of tongue tissues from sentinel rats and 4-nitroquinoline 1-oxide (4NQO) exposed rats fed control diet, 5% black raspberry (BRB), 10% BRB, or 0.4% ellagic acid (EA). Paraffin sections were stained with anti-Ki-67 antibody and counterstained with hematoxylin. **(B)** Quantitation of Ki-67 positive cells in tongue epithelial tissue from sentinel rats as well as 4NQO exposed rats fed with control diet, 5% BRB, 10% BRB, or 0.4% EA. Graphic representation of percent Ki-67 positive nuclei relative to total nucleated cells in six random fields of tongue epithelium from selected rats from each experimental group. Data are expressed as percent cells ± SE. *p-value < 0.05 for each group comparison using independent Student’s *t*-test. **(C)** Gene expression profiles of the cell cycle associated biomarkers *Aurka, Ccna1*, and *Ccna2* during 4NQO-induced oral carcinogenesis as determined by reverse transcription quantitative PCR. Data are presented as mean fold induction over sentinels ± SE. *p-value < 0.05 for each group (*N* ≥ 20) comparison using analysis of variance analysis.

Previous studies demonstrate the ability of BRB phytochemicals to inhibit cancer cell proliferation and promote apoptotic pathways in oral cancer cells *in vitro* (8, 9). These results are supported by transcriptional analysis of proliferative and apoptotic genes in clinical trials of oral cancer patients (15, 21, 28). We therefore analyzed the effects of dietary administration of BRBs on proliferative and apoptotic pathways during experimental oral carcinogenesis in our rat model. To characterize BRB-mediated effects on proliferative pathways, we analyzed the expression of Ki-67 (a proliferation marker) in tongue lesions using IHC. Proliferative indices were significantly reduced in carcinogen exposed mice that received 5 or 10% BRBs (Figures 5A,B). Further analysis of proliferative biomarkers by RT-qPCR analysis of rat tongues demonstrated a reduction in gene expression of the cell cycle regulated kinase *Aurka* which has been shown to play a role in tumor development and progression (Figure 5C). Other cell cycle associated genes inhibited by BRBs include *Ccna1*, which was inhibited by 5% BRBs and *Ccna2*, which was inhibited by 5 and 10% BRBs (Figure 5C). Our results demonstrate that dietary administration of BRBs inhibits cellular proliferation in tongue lesions during 4NQO-induced oral carcinogenesis.

**Figure 6:**
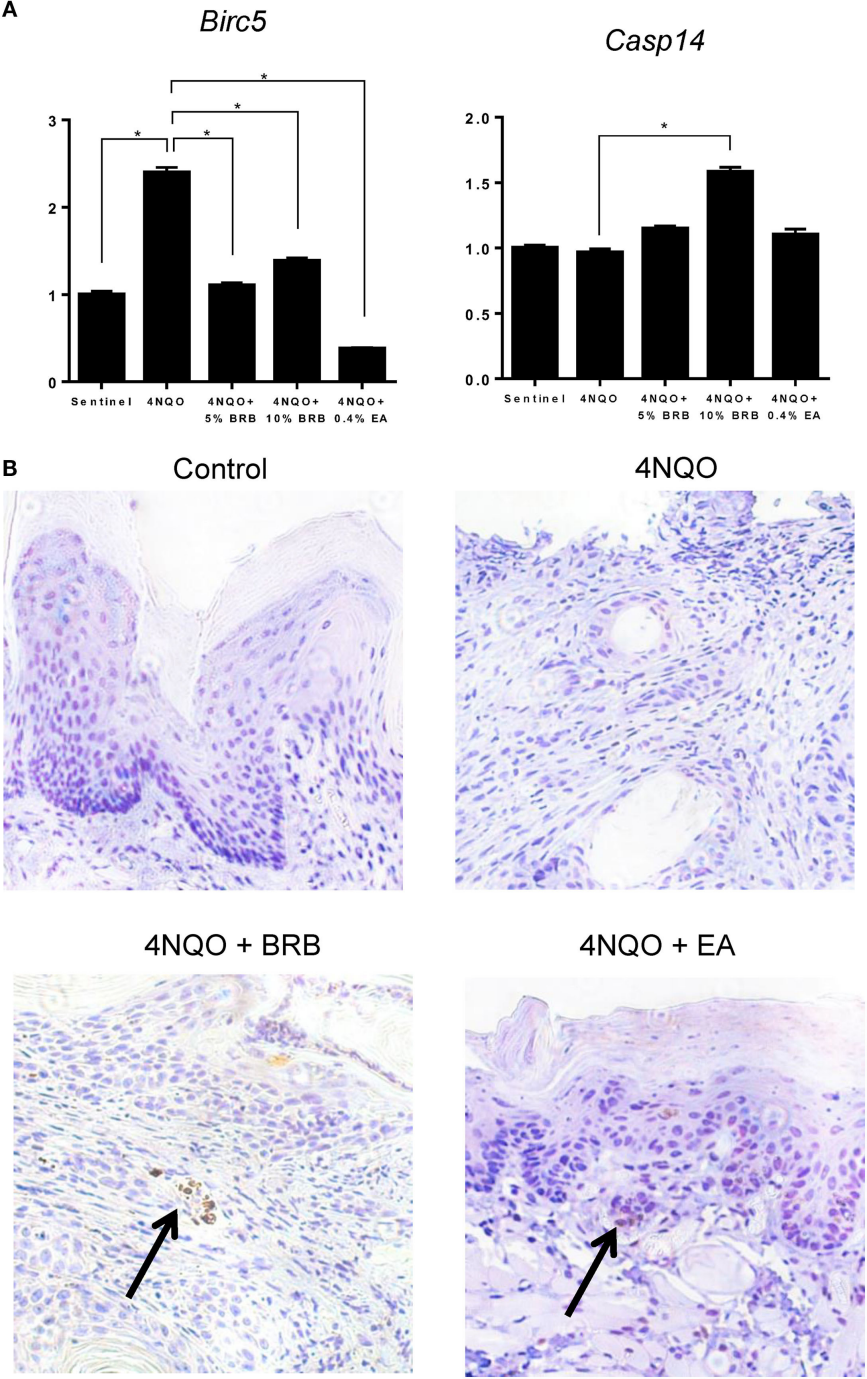
**(A)** Gene expression profiles of the apoptotic biomarkers *Birc5* and *Casp14* during 4-nitroquinoline 1-oxide (4NQO)-induced oral carcinogenesis as determined by reverse transcription quantitative PCR. Data are presented as mean fold induction over sentinels ± SE. *p-value < 0.05 for each group (*N* ≥ 20) comparison using analysis of variance analysis. **(B)** Representative immunohistochemistry stained microscopic images (×100) of tongue tissues from sentinel rats and 4NQO exposed rats fed control diet, black raspberry (BRB) supplemented diet, or ellagic acid (EA) supplemented diet. Paraffin sections were stained with antibodies against cleaved caspase 3 and counterstained with hematoxylin. In 4NQO-exposed sections, regions of dysplasia or squamous cell carcinomas are shown. Arrow points to positively stained (apoptotic) cells.

Next, we determined the effect of BRB administration on apoptotic pathways during oral carcinogenesis. Gene expression analysis by RT-qPCR demonstrated that *Birc5* (survivin), a member of the inhibitor of apoptosis (IAP) family, was downregulated by BRB treatment (Figure 6A). *Birc5*, which is transcriptionally upregulated during squamous cell carcinomas, exerts its function by inhibiting caspase activation, thereby blocking apoptosis (29). Targeting *Birc5* is therefore a promising strategy for oral cancer therapy. Inhibition of *Birc5* gene expression by BRB, led us to examine its effect on its downstream target caspase-3. We therefore examined cleaved caspase 3 protein expression in tongue tissues of 4NQO-treated and BRB-treated rats by IHC. Our results show that in squamous cell carcinomas, cleaved caspase 3 expression is increased in BRB-treated tissues (Figure 6B). We also observed that gene expression of *Casp14*, an apoptotic and keratinocyte senescence biomarker, was slightly upregulated in 10% BRB-treated rat tongues (Figure 6A). Taken together, our molecular analysis of experimental rat oral carcinogenesis suggests that modulation of apoptotic pathways is a mechanism of BRB-mediated oral cancer chemoprevention.

### EA Inhibits Tumor Incidence but Does Not Reduce the Formation of Premalignant Lesions

Ellagic acid, a bioactive component of BRBs as well as other fruits, nuts, and vegetables, is a naturally occurring antioxidant and anti-proliferative polyphenolic compound known for its anticancer activities in preclinical studies (17, 30, 31). We used this compound as a control bioactive substance to compare to the oral cancer inhibitory activities of BRBs in our rat 4NQO oral cancer model. Surprisingly, as shown in Table 1, although EA reduced tumor incidence (0.03 EA vs 0.18 4NQO), multiplicity of premalignant lesions in EA treated rats were higher compared to 4NQO-only induced rats (4.18 EA vs 3.39 4NQO).

Gene expression profiles of 4NQO exposed EA treated rats demonstrate a reduction of genes associated with inflammation (*Il-1β, Cxcl1, Mif, Nfkb1, Arg1, Ptgs1*, and *Ptgs2*) (Figure 4A), cell proliferation (*Aurka, Ccna1*, and *Ccna2*) (Figure 5C), and apoptosis (*Birc5*) (Figure 6A). Serum levels of Cox2 (Figure 4B), and Ki-67-based cellular proliferation (Figure 5B) were also reduced in EA treated rats. Taken together, our data show that although EA inhibited the formation of malignant lesions in the rat tongue and modulated biomarkers of molecular efficacy, it is not as effective as BRBs in preventing the development of premalignant lesions during experimental rat oral carcinogenesis. Nevertheless, the potential of EA in adjuvant or complementary therapeutic applications against oral carcinogenesis is clearly demonstrated by the anti-oral cancer effects of EA observed in this study.

## Discussion

Oral squamous cell carcinoma is the resultant endpoint of an accumulation of genetic alterations that permit sequential transition from phenotypically normal cells to varying grades of dysplasia and invasive disease. The exposure of the entirety of the oral cavity by carcinogens initiates a field of epithelia with genetic defects at risk for progression to malignancy, vis-à-vis field cancerization. This presents a wide range of opportunities for chemoprevention by BRBs as previous preclinical and clinical studies have demonstrated. However, in order to extend the application of BRB phytochemicals in oral cancer chemoprevention, a suitable animal model that recapitulates the fundamental features of human oral cancer and facilitates the characterization of the cellular and molecular changes associated with BRB-mediated oral chemoprevention is essential. Although the hamster model sufficiently demonstrates the efficacy of BRBs in the chemoprevention of oral carcinogenesis (10, 11), extended investigation on the specific mechanisms of oral chemoprevention presents challenges due to the limited availability of molecular resources using this model. The rat 4NQO model of oral carcinogenesis and chemoprevention by BRBs, as described in this study, represents an essential model for demonstrating the molecular efficacy of BRBs in inhibiting oral carcinogenesis, and for determining mechanisms of BRB-mediated oral cancer prevention.

The molecular changes observed during rat 4NQO oral carcinogenesis and BRB-mediated oral chemoprevention very well mimic those observed during an oral cancer chemoprevention trial using a BRB phytochemical-rich troche (15). Key molecular pathways modified by BRB phytochemical intervention in this recent chemoprevention clinical trial include genes regulating inflammation (*NFKB1* and *PTGS2)* and cell survival (*AURKA* and *BIRC5*). In a similar manner, these genes were significantly reduced by BRB administration in our rat model. Therefore, we can reasonably conclude that this model validates the BRB-mediated transcriptional signature in human oral squamous cell carcinomas (15).

Our results identify pro-inflammatory pathways as a major target of BRBs during oral cancer chemoprevention. A chronic pro-inflammatory microenvironment is essential to the establishment and progression of oral cancer. Inflammation contributes to oral carcinogenesis by causing the release of factors that promote cell growth, proliferation, angiogenesis and invasion (25). Furthermore, the release of inflammatory by-products, such as reactive oxygen species, accelerates mutagenic events that contribute to malignancy. Not surprisingly, therapeutic strategies that target pro-inflammatory pathways in oral cancer are the focus of extensive research efforts. Pro-inflammatory mediators which were inhibited by BRB phytochemicals during experimental rat oral carcinogenesis in our study include *Nfkb1, Ptgs2, Il-1β, Cxcl1, Mif*, and *Nfe2l2*. Similar to our previous Phase 0 BRB oral chemoprevention trial, expression of *Nfkb1* was inhibited by 5% BRB and 0.4% EA administration to oral cancer induced rats. *Nfkb1* mediates a chronic pro-inflammatory tumor microenvironment and constitutes a missing link between inflammation and cancer (32, 33). Inhibition of NF-κB signaling has been associated with reduction of inflammation-associated cancers (33). The effect of BRBs on NF-κB inhibition is especially relevant to human oral carcinogenesis, given that major risk factors for oral cancer (tobacco smoking, alcohol consumption, and HPV infection) are linked to chronic inflammation. *Ptgs2* is a major enzyme in the prostaglandin biosynthesis pathway and an inflammatory biomarker that is known to contribute to oral carcinogenesis. Although *Ptgs2* expression was inhibited in tongues of oral cancer induced rats administered 5% BRB or 0.4% EA, we did not observe any differences in the levels of Cox-2 in the sera of these rats. This suggests that the main effects of BRBs are localized to the oral epithelia of these rats and that systemic effects are limited. Although some BRB metabolites are detectable systemically (34), it is likely that the major anti-inflammatory effects of the bioactive compounds in BRBs are localized to the tissues of administration (mucosa), which makes this chemopreventive agent most impactful against cancers of the oral and gastro-intestinal mucosae (11, 12, 15, 35).

We recently demonstrated that genetic deletion of *Mif*, another pro-inflammatory mediator linking inflammation and cancer, inhibits experimental oral carcinogenesis (26). *Mif* signals through the ERK1/2 MAP kinase signaling pathway, leading to the expression of other pro-inflammatory cytokines such as *Il-1β* and *Cxcl1*. We therefore investigated the impact of BRBs on *Mif* expression and targets of this signaling pathway. Our data established that *Mif, Il-1β*, and *Cxcl1* expression were inhibited by bioactive components in BRBs, including EA. These pro-inflammatory mediators are crucial to the recruitment of innate immune cells to the oral tumor microenvironment, where they contribute to oral tumor progression.

Inhibition of apoptosis is a common strategy that enables cancer cell survival and progression (25). Increased expression of the anti-apoptotic regulator Birc5 (survivin) is one mechanism by which oral cancer cells resist apoptosis (29). In our study, there was a significantly increased expression of *Birc5* mRNA in untreated, 4NQO exposed rats. BRB administration significantly inhibited *Birc5* gene expression. Further, we demonstrated a concomitant increase in cleaved caspase-3 protein expression in oral lesions of BRB-treated rats. Our analyses of the molecular events that occur during BRB-mediated chemoprevention of experimental oral carcinogenesis demonstrate that targeting of the apoptotic pathway through inhibition of *Birc5* gene expression is a potential anticancer mechanism of BRBs.

Evidence for inhibition of proliferative signaling as a mechanism of BRB-mediated chemoprevention of oral cancer is demonstrated by the downregulation of cell cycle associated genes that are typically associated with oral carcinogenesis: *Aurka, Ccna1, Ccna2*, and *Ccnd1* (15, 27). This is further supported by the reduction in the expression of the proliferative marker Ki-67 in BRB-treated, carcinogen-induced rats. Of particular interest is *Aurka*, a serine/threonine kinase which plays an important role during G2 to M phase transition, is involved in various mitotic events such as centrosome maturation and separation, mitotic entry, spindle assembly, and chromosome alignment (36). Selective inhibitors of AURKA have been developed for the treatment of solid tumors and have been tested in preclinical studies against oral cancer (37). The observed effects of BRBs on this key mitotic regulator as well as in other cell cycle associated pathways demonstrates the efficacy of BRBs as a whole-food additive or synergistic mixture of complementary phytochemicals targeting multiple oncogenic pathways in oral cancer chemoprevention and therapy.

It is noteworthy that in our study 5% BRBs was more effective than 10% BRBs in inhibiting oral lesion development as well as in reducing molecular biomarkers of oral carcinogenesis. This is not entirely surprising given that similar results were observed in the hamster check pouch model of oral carcinogenesis (10) as well as rat models of esophageal and colon carcinogenesis (38). It does underscore the importance of dosage in the application of BRB-mediated oral cancer chemoprevention strategies. The complex mixture of phytochemicals in BRBs participates in a complex network of interactions within the tumor microenvironment, which ultimately affects the optimal dosage for oral chemopreventive efficacy. Future studies should address this complex network of interactions between BRB phytochemicals and the oral tumor environment, as well as the effect of phytochemical combinations on the chemopreventive efficacy of BRB. This will provide essential knowledge that will be useful in the design and implementation of future clinical trials on BRB oral cancer chemoprevention.

A major bioactive component of BRBs with demonstrable anticancer activity is EA. Numerous studies demonstrate the anticancer activity of this dietary polyphenol using *in vitro* (39) and *in vivo* (17, 30, 31) oral cancer models through mechanisms involving apoptotic (31), angiogenic (30), and proliferative (39) signaling pathways. We also found evidence for the modulation of some of these pathways in our transcriptional analysis. However, an interesting finding from our study was that while EA significantly inhibited malignant lesion development, premalignant lesions were not inhibited in EA-treated rats compared to untreated rats. Surprisingly, the incidence of gross premalignant lesions (erythroplakia and leukoplakia) was higher in EA treated rats compared to untreated rats. Taken together, these observations suggest that, in addition to pro-inflammatory, anti-apoptotic, and cell cycle associated pathways, other mechanisms are involved in BRB-mediated inhibition of oral carcinogenesis. This is not surprising, since BRB contains a complex mixture of phytochemicals that could target other oral cancer pathways that are not described in this study. Interestingly, a previous study by Tanaka et al. showed that in 4NQO-induced rats oral carcinogenesis, 0.4% EA administration significantly inhibited the development of tongue tumors as well as hyperplastic and dysplastic lesions (17). It should be noted that in the Tanaka et al. study, rats were given 4NQO for 5 weeks and EA administration began one week prior to 4NQO exposure and continued until 1 week after 4NQO exposure. In our study, rats were exposed to 4NQO for 14 weeks and EA administration began after 4NQO exposure. This difference in the sequence and length of 4NQO exposure coupled with the differences in the time of administration of EA likely accounts for the substantive difference between these studies. While the chemoprevention model employed by Tanaka et al. is adequate for screening biologically active compounds, it has only minimal relevance to the exposure-risk reduction paradigm required for human chemoprevention studies. It is evident that the protective effect of EA depends on where along the multistep process of oral carcinogenesis intervention begins; a factor that must be considered in determining the most appropriate approach to oral cancer chemoprevention. We believe that our animal model more accurately recapitulates the exposure-cancer progression paradigm with chemopreventive strategy initiated after identification of individuals at high risk for the development of oral cancer. Furthermore, it is not surprising that BRB administration was more effective than EA in reducing the development of premalignant lesions in our model due to the complex milieu of bioactive phytochemicals that may act in an additive or synergistic manner to inhibit oral cancer development. This supports the notion that a combination of bioactive compounds, such as is found in whole foods, is potentially more effective against oral cancer.

In summary, we show that dietary administration of BRBs inhibits gross and histopathological lesion formation during 4NQO-induced rat oral carcinogenesis. This was associated with a reduction in pro-inflammatory, anti-apoptotic, and proliferative molecular biomarkers. Our results also recapitulate the predictive biomarker signature observed in a human oral cancer chemoprevention clinical trial using BRB troches (15). Further, our experimental oral cancer model shows that modulation of pro-inflammatory, apoptotic and cell cycle associated pathways are potential mechanisms of BRB-mediated oral cancer chemoprevention. Combined with data from previous studies, our results demonstrate that the incorporation of BRB phytochemicals in oral cancer chemoprevention and complementary therapy is a potentially viable strategy for oral cancer prevention and treatment.

## Ethics Statement

This study was carried out in accordance with the recommendations of Ohio State University Laboratory Animal Resources guidelines, Ohio State University Institutional Animal Care and Use Committee. The protocol was approved by The Ohio State University Institutional Animal Care and Use Committee.

## Author Contributions

SO, CW, and TK designed the study. SO, TK, BC, LW, JM, PG, KH, CB, BW, KS, and TK performed experiments and acquired data. SO, JA-J, CW, and TK analyzed and interpreted data. SO and TK drafted the manuscript. All authors critically revised and approved the final manuscript.

## Conflict of Interest Statement

The authors declare that the research was conducted in the absence of any commercial or financial relationships that could be construed as a potential conflict of interest.
